# The ghost of infections past: Accounting for heterogeneity in individual infection history improves accuracy in epidemic forecasting

**DOI:** 10.1371/journal.pbio.3003311

**Published:** 2025-08-11

**Authors:** Pedro F. Vale, Chadi M. Saad-Roy, Mike Boots

**Affiliations:** 1 Institute of Ecology and Evolution, School of Biological Sciences, University of Edinburgh, Edinburgh, United Kingdom; 2 Miller Institute for Basic Research in Science, University of California, Berkeley, California, United States of America; 3 Department of Integrative Biology, University of California, Berkeley, California, United States of America

## Abstract

Variation in infection history is an important but often underappreciated driver of individual variability in responses to infections. Such individual heterogeneity in immune responses, stemming from variable previous exposure to pathogens, subsequently influences epidemiological outcomes. By comparing research on innate immune priming in invertebrates, which lack adaptive immune memory but demonstrate enhanced responses to re-infections, to patterns seen in vertebrates, this Essay reveals broad implications for disease dynamics. Insights from mathematical modelling and experimental data highlight the critical need to integrate evolutionary disease ecology into public health initiatives to better predict and manage infectious diseases.

## Abstract 2

Variable pathogen exposure history contributes to individual immune differences, complicating epidemic forecasting. This Essay argues that experimental disease ecology can offer powerful tools and approaches to better understand and predict the epidemiological consequences of variable infection history.

## Introduction

Understanding the dynamics by which infections persist or are cleared by hosts under different immune scenarios can substantially influence public health strategies for managing infectious diseases [1–3]. A significant barrier to this understanding is individual host heterogeneity arising from a variety of genetic and environmental sources of variation, which impact multiple traits that determine the likelihood of acquiring and transmitting infections [4–6]. A common, but infrequently measured source of individual heterogeneity is the initial immune state, which is determined by the unique infection history of each individual [2,7,8]. Variation in the initial immune state is relevant because re-exposure and re-infections (or cross-immunity from other pathogens) are common for many infectious diseases [9–11], and the epidemiological outcomes of re-infections are likely to vary between individuals according to their genetic background and the prior level and duration of pathogen exposure [12–14]. Infection history therefore creates immune uncertainties, complicating epidemiological projections. For example, as the COVID-19 pandemic progressed, mathematical models were used to illustrate the large ranges of potential immuno–epidemiological trajectories caused by underlying immune uncertainties [15]. Thus, understanding infection history, immune responses and how they shape host–pathogen dynamics is critical.

The epidemiological consequences of infection history and the resulting differences in host immune state have been largely addressed theoretically, by modelling epidemic outcomes that assume some level of heterogeneity in susceptibility [1,12–18]. However, the difficulty in measuring population variation in immune state has meant that theoretical explorations of its epidemiological consequences far outpace the empirical tests of theoretical predictions [4,7]. In this Essay, we focus on the issue of heterogeneity in susceptibility arising specifically from variable infection histories and link it to separate but highly relevant work carried out by evolutionary disease ecologists on a variety of non-human host species. Notably, although vaccination can also lead to strong heterogeneity in immune states before infection [3], and vaccination strategies such as ‘prime–boost’ vaccination are commonly used to enhance immunological responses [19], in this Essay we focus instead on the effects of prior pathogen exposure.

**Fig 1:**
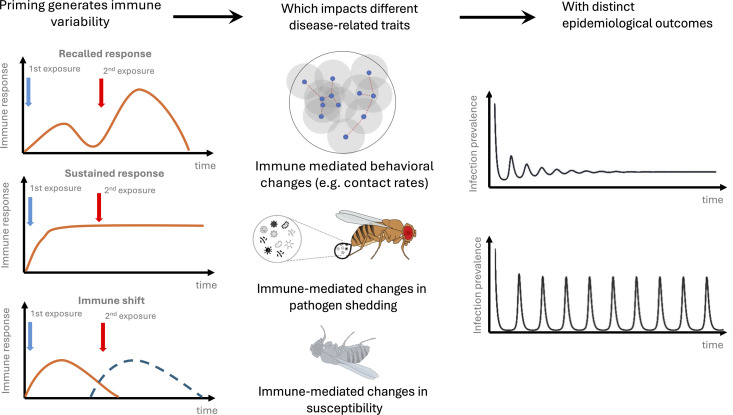
How variation in infection history generates heterogeneity in epidemic outcomes. Following an initial pathogen exposure, individuals experience distinct immune responses upon re-infection (left panel). In some cases, this may be an infection that is cleared. However, it is also important to consider infection histories where exposure (but not necessarily infection) has occurred, which could equally lead to subsequent changes in epidemiological outcomes upon re-exposure. These responses may differ between host species and according to the identity, strength and duration of the pathogen exposure [39]. For example, in response to a primary pathogen exposure (blue arrows), an immune response is mounted (orange line), such as the expression of antimicrobial peptides (AMPs). Following a secondary exposure (red arrow), the initial exposure may result in a stronger AMP response (recall response [22]), a sustained AMP response that remains elevated upon secondary exposure (sustained response [40]), or result in a shift to a distinct kind of immune response, such as a cellular response (dashed blue curve; an immune shift [41]). These immune shifts will in turn impact the mean and the variance of disease-related traits (middle panel), such as changes in host behavior that will impact the contact rate between individuals (e.g., sickness behaviors [42,43]), or immune mediated changes in pathogen shedding and susceptibility [20,44,45]. A consequence of these changes in disease-related traits is generally an increase in trait heterogeneity among individuals, which will impact pathogen transmission (right panel), with markedly different epidemiological outcomes [25–27].

We aim specifically to derive insights from work on the epidemiological consequences of innate immune priming in invertebrates. Although invertebrates have not evolved the same ‘adaptive’ B cell and T cell responses that result in specific immune memory in vertebrates, there is abundant evidence that in many invertebrate species, prior exposure to pathogens can prime the innate immune system, leading to stronger responses upon subsequent infections [20–24] (Box 1). Theoretical and experimental work has highlighted that invertebrate immune priming can profoundly affect epidemiological outcomes by reducing susceptibility and transmission potential, but that the extent of this impact strongly depends on the initial priming conditions and the nature of the pathogen exposure [20,24–27]. Importantly, theoretical predictions regarding the epidemiological outcome of variable infection history arise solely from the effect of prior exposure on key disease-related traits (such as susceptibility, infectiousness and contact rates; Fig 1), and do not depend on the specific immune mechanisms underlying the priming response, which vary widely among vertebrates and invertebrates (Box 1). Clear and common themes therefore emerge between work exploring the epidemiological consequences of individual heterogeneity in immune state for human infections, and similar consequences of variation in immune priming in invertebrates.

**Box 1. Divergent immune responses can lead to similar infection outcomes.**

Insects have evolved a sophisticated immune system that relies primarily on innate immunity, as they lack the adaptive immune response found in vertebrates [28]. Broadly, insect immune responses include cellular responses (phagocytosis, encapsulation and melanization) and humoral responses (antimicrobial peptide production) [29]. Upon infection, pathogens are recognized by pattern recognition receptors (PRRs) that detect pathogen-associated molecular patterns. This triggers signaling pathways such as the Toll and Imd pathways, leading to the nuclear translocation of NF-κB-like transcription factors and subsequent expression of antimicrobial peptides, which are secreted to combat the pathogens [30–32].

Immune priming in invertebrates has been particularly well-explored in many insect species and refers to a phenomenon in which prior exposure to a pathogen or its components results in improved survival upon subsequent encounters [21,33]. This survival benefit is often the consequence of an enhanced immune response triggered by the initial pathogen exposure. Specific immune priming mechanisms vary across species but generally involve heightened expression of immune-related genes, increased antimicrobial peptide production, and enhanced activity of immune cells such as hemocytes [21,24]. For example, in crustaceans such as the woodlouse, prior exposure to a pathogen can lead to faster encapsulation and melanization responses upon re-infection [34]. Similarly, insects such as *Drosophila* have increased resistance to secondary infections through epigenetic modifications and other regulatory mechanisms that affect gene expression involved in both cellular and humoral immune defenses [20,23,35,36].

By comparison, mammals have evolved both innate and adaptive immune systems [37]. The innate immune system shares mechanisms such as phagocytosis and the general use of PRRs with insects. However, vertebrates have evolved an adaptive system characterized by lymphocytes (T cells and B cells) that provide memory and specificity to the immune response through mechanisms such as antibody production and antigen presentation [37]. This immune memory is highly specific and relies on the recognition of specific pathogen-derived antigens. Upon initial exposure, antigen-specific lymphocytes proliferate and differentiate into memory cells, which persist long-term and can mount rapid and potent responses when re-exposed to the same antigen. This process is central to the effectiveness of vaccines in humans and other mammals [38].

Our definition of priming is intentionally broad, as any exposure (that may or may not result in infection) can lead to changes in disease-related traits and therefore has the potential to modify epidemiological outcomes. While the mechanisms that prime the immune response for subsequent pathogen exposure in both invertebrates and vertebrates are fundamentally different, a strong body of evidence supports the ideas that invertebrates can exhibit a form of immune priming that results in a broad, nonspecific enhancement of the innate immune system.

In this Essay, we describe some of this work, draw on the similarities between invertebrates and vertebrates and derive some general insights. We first describe mathematical modelling that explores the epidemiological consequences of invertebrate immune priming and highlight recent work that models the epidemiological impact of the initial immune state in vertebrate immunity. We then turn our focus to recent experimental work in both vertebrates and invertebrates, where the impact of pathogen priming on different disease-related traits is quantified. We finish by outlining some important outstanding research questions and discuss how invertebrate model systems could be utilized to make addressing these questions more tractable.

### What does theory predict?

A substantial body of theoretical work has made use of epidemiological models to explore the impact of prior exposure (or infection) on key epidemiological traits [1,12–18]. Much of this work has been motivated by immune priming in invertebrates, but there are also examples of models that explore the epidemiological consequences in vertebrates with adaptive immune responses (Box 1). In this section, we describe a few key examples that help illustrate the importance of accounting for infection history for accurate epidemic predictions.

#### Insights from invertebrate immune priming models.

One of the earliest theoretical explorations of how invertebrate immune priming impacts the epidemic spread of infection focused on how it affects pathogen persistence and host population dynamics [27]. By deriving a susceptible–primed–infectious (SPI) model, this work contrasts with the classic susceptible–infectious–recovered (SIR) framework, by considering how low doses of pathogens that do not cause infection can prime the immune response, potentially leading to increased protection against future infections. The key epidemiological effect is that this protection occurs in individuals that have never been infectious, which contrasts with the classic characterization of protection as adaptive immunity in individuals that have recovered after having been infectious (SIR). This work was important in establishing that immune priming can lead to destabilization of host populations, especially under high rates of priming that do not result in full immunity. Specifically, the model predicts that high levels of immune priming could lead to significant oscillations in population dynamics through the generation of limit cycles, the extent of which depend on the proportion of susceptible individuals that become primed, the level of protection on future exposure that immune priming provides and, importantly, the reproductive costs to the host of priming and infectiousness. Immune priming was also shown to reduce the effective reproduction number (*R*_0_) of a pathogen, thereby constraining disease invasion and persistence. Immune priming can therefore influence disease dynamics in important ways, not only enhancing individual protection but also introducing potential instability into population dynamics.

Another model of priming addressed how immune priming impacts the dynamics of disease spread and maintenance across populations of invertebrates that were subject to different modes of pathogen transmission [26]. By modelling both direct continuous transmission and obligate killer dynamics (in which transmission happens only when infected individuals die), the authors found that immune priming could either stabilize or destabilize populations depending on the mode of transmission. In directly transmitted infections, the stability of the disease-free equilibrium was attainable when priming resulted in a lower transmission rate, a higher recovery rate or a higher disease-induced mortality rate. For obligate killer (and non-human) transmission systems, the model suggested more complex dynamics, with stability dependent on a combination of disease-induced mortality, decay rates of infectious cadavers and recovery rates. Notably, cadaver decay has a critical role, as higher cadaver decay rates reduce the duration that a cadaver remains infectious, thus impacting the spread of the pathogen. Additionally, this work highlighted how the effect of priming on the disease-induced mortality rate had a more significant effect on the likelihood of disease-free equilibrium stability compared with direct transmission. Overall, these findings show that immune priming has a significant and variable role in influencing the spread and persistence of infectious diseases, and that this role depends heavily on the mode of pathogen transmission.

Such SPI models have also been integrated into a dose–response framework, which enables the exploration of how varying levels of immune activation based on previous exposures shape within-host dynamics and transmission characteristics [25]. This approach has shown that priming could, under certain conditions (for example, when immunity is ‘complete’ or ‘leaky’), inflate disease prevalence, especially in direct transmission scenarios. This result emerges because primed individuals, although less likely to die from the infection, might still transmit the pathogen (if immunity is incomplete). The type of immunity conferred by priming (whether it is ‘leaky’ immunity, in which primed individuals gain partial protection, or ‘all-or-nothing’ immunity, in which only some are fully protected) therefore determines the epidemiological outcome. Immune priming can therefore act as a double-edged sword by potentially increasing disease prevalence through mechanisms analogous to leaky vaccinations.

#### Modelling mammalian immune responses.

The studies discussed above were motivated by a desire to understand the epidemiological consequences of invertebrate immune priming [25–27]. By contrast, a recent study focused on modelling a mammalian immune system with an explicit interaction between parasite biomass and T-helper-cell-mediated immune responses [46]. The model included key immunological feedback mechanisms, such as cytokine production and mutual inhibition among T helper cell subpopulations, to explore how immunological feedback loops can generate parasite persistence thresholds and explain the variation in infection duration within hosts.

Although the impact of prior exposure or priming (in the invertebrate sense) was not the focus of the work, the model showed that the initial immune state of the host and the dose of parasite critically determined whether an infection would be cleared or persist. The model predicts that strong immunological feedback loops can lead to multiple stable immune equilibria, implying that slight changes in initial conditions can lead to divergent outcomes in terms of infection duration and severity. Notably, the study demonstrated that chronic infections can still occur despite a strong and effective immune response if initial conditions and parasite characteristics are conducive. In the context of individual heterogeneity in the initial immune state, this work nicely highlights that immunological feedback loops are intrinsic to how immune responses are regulated and can lead to significant variability in infection outcomes based on initial conditions.

While prior infection can lead to host immunity, infection history may still matter even if a pathogen is not immunizing. For example, a recent theoretical model revealed that mortality after recovery (i.e., post-infection mortality; PIM) can result in sustained epidemiological oscillations [47]. In that work, the authors formulated a model that considered two types of susceptible individuals: those who have never been infected and those who have been previously infected but have recovered. In the latter group, long-term effects of pathogens may lead to slightly elevated rates of mortality. If immunity upon recovery is not robust, PIM can be destabilizing and result in sustained epidemiological oscillations. This effect emerges because of the interaction between PIM and the susceptible individuals who have been previously infected (i.e., they experience higher mortality, which depletes the pool of susceptible individuals and consequently leads to a decline in infections, thus enabling the build-up of never-infected susceptible individuals). By contrast, robust immunizing responses (modelled as a decrease in relative susceptibility to reinfection) are stabilizing, as recovered individuals contribute negligibly to the pool of susceptible individuals. While this theoretical model does not address priming in the invertebrate sense that we describe above, it nevertheless highlights the importance of characterizing infection history to understand pathogen population dynamics.

#### What can we learn from experimental disease ecology?

In the previous section, we highlighted how prior infection can generate individual heterogeneities that may have important and sometimes counterintuitive epidemic outcomes. To make accurate epidemic predictions, it is therefore essential to have information on the extent of the variation in the initial immune state, which is often impacted strongly by individual infection history.

Recent work has investigated how prior pathogen priming affects the transmission potential and outcomes of reinfection in hosts by using house finches and the bacterial pathogen *Mycoplasma gallisepticum* as models [44]. Specifically, the study assessed whether previous exposure to *M. gallisepticum* influenced the likelihood and efficiency of transmitting the pathogen during subsequent infections with either a homologous or a heterologous strain. House finches were challenged with three different priming conditions (none, intermediate, using repeated low-dose exposures, or high, using a single high-dose exposure) with the *M. gallisepticum* strain. Following a recovery period, birds were reinfected with either a homologous or a heterologous strain of *M. gallisepticum* and then housed with a naive cage mate to assess transmission success. This work found that higher levels of priming (particularly the high priming condition) resulted in reduced disease severity and lower pathogen loads during subsequent reinfections. The degree of priming also significantly impacted transmission success, with the highest priming level showing the strongest reduction. Overall, this study shows that prior exposure to a pathogen significantly reduces the likelihood of transmission during subsequent reinfections, although highly virulent strains could overcome this protection.

Other work in the same system addressed how prior exposure to a bacterial pathogen affects heterogeneity in susceptibility among house finches, and the subsequent epidemiological consequences of this heterogeneity [45]. Here too, house finches were exposed to varying doses of a bacterial pathogen (none, low-dose or high-dose). This work showed that birds with prior exposure to the pathogen exhibited a greater heterogeneity in susceptibility compared with naive birds, with those exposed to higher doses showing the most variability. Using an epidemiological model to simulate epidemic outcomes under different scenarios of heterogeneity in susceptibility, the authors found that this variability significantly impacted the size of disease outbreaks, with greater heterogeneity correlating with smaller epidemic sizes. Another finding was that prior exposure also reduced infection-induced mortality, which has implications for population dynamics and disease management. Prior exposure to pathogens may therefore significantly influence the heterogeneity of susceptibility within host populations, which can subsequently have important effects on epidemic dynamics, particularly in reducing the size of outbreaks. These results generally echo theoretical findings on variation on host susceptibility [1,48,49].

Moving to a different experimental model, recent work in *Drosophila* revealed that flies that had previously been exposed to an inactivated form of the bacterial pathogen *Providencia rettgeri* showed lower mortality than unprimed flies, driven by a transient decrease in pathogen loads [20]. This reduction had important epidemiological consequences, as primed flies showed lower levels of pathogen shedding at the individual level and caused less severe epidemics at the population level compared to unprimed flies. Behavioral traits that could be epidemiologically relevant, such as the activity level (a proxy for contact rate), were not affected by priming, although other work has described changes in the activity [50–52] and social aggregation [42,53] of flies following infection. This result highlights a benefit of invertebrate systems, which are often amenable to population-level assays, including transmission assays, making them feasible tools to use to link variation at the individual level to changes in experimental epidemics, which would not be tractable for most animal systems [20,54–56].

### Model systems for studying the effects of infection history

Many theoretical predictions may prove difficult to test in some experimental systems, particularly when the effect of prior exposure must be measured on multiple behavioral and physiological host traits. While primed individuals may live longer, thus extending the infectious period, their pathogen burden may be lower which, depending on the degree of protection and the extent to which this increases the infectious period, could either lead to lower or higher pathogen shedding and thus to more or less severe epidemics. Further, behavioral changes in primed individuals may also be likely, if immune deployment during the initial exposure leads to sickness behaviors such as lethargy or reduced social interaction [42,43,53].

**Fig 2:**
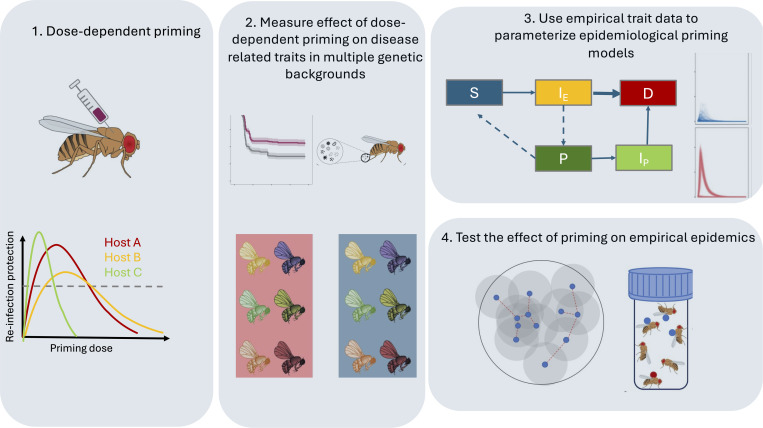
An empirical framework to address the epidemiological consequences of variable infection history. Epidemic modelling predicts that the epidemiological effects of prior pathogen exposure depend on the degree of protection conferred by priming, which can impact the extent to which it affects the pathogen’s colonization success and the host’s ability to clear or tolerate the infection. In this figure, we use the fruit fly *Drosophila melanogaster* as an illustrative example of how the epidemiological consequences of variable infection history can be addressed empirically. A fundamental first step is to measure a dose-response of prior exposure on relevant disease related traits (panel 1). Thus, Host A, Host B and Host C refer to 3 hosts with different phenotypic expression of such disease related traits. A further level of complexity arises from genetic variation in the expression of each of these host traits (panel 2). Another important consideration when addressing the epidemiological effect of infection history is to quantify how prior exposure (*i.e.,* the pink or blue of the boxes) impacts the expression of multiple disease-related traits, and how this expression is itself genetically variable among hosts (different colors of flies within each box). Once such data is acquired in controlled lab settings, it becomes possible to explore the epidemiological outcomes in two distinct but complementary ways, by using the data to parameterize system-specific epidemiological models that explicitly account for phenotypic differences between susceptible (S), infected exposed (I_E_), primed (P) and primed re-infected hosts (I_P_) (panel 3). The other approach (panel 4) is to design experimental epidemics (on a specific contact network, e.g., left-half of this panel) choosing hosts with known combinations of traits (e.g., red and blue circles on flies in the vial schematic), in ways that enable the genetic and trait-based contributions to epidemic dynamics to be disentangled [62,63]. Some elements have been modified from [20], published under a CC-BY license.

The need to measure trait-specific responses to variable immune exposure highlights two major outstanding questions: how does prior infection history modify different disease-related traits; and, in which traits does heterogeneity matter most for epidemic outcomes? For vertebrates, such as humans, variation in the strength and duration of immune responses following recovery (due to the adaptive immune system) can complicate epidemiological dynamics [57]. However, we argue here that invertebrate host–pathogen systems are tractable and powerful systems that can be used to explore how individual level heterogeneity arising from different infection histories could scale up to population-level epidemiological outcomes [6,18,45] (Box 2, Fig 2). *Caenorhabditis elegans* nematodes [56,58], *Drosophila* fruit flies [6,20] and *Tribolium* beetles [59,60] are particularly promising host systems in which priming has clear effects on important epidemiological traits and may even determine the evolutionary trajectory of pathogens [58]. A key advantage of these systems is the possibility for high throughput measurement of these traits in multiple genotypes or genetically variable, wild-derived populations [21,56,60,61].

**Box 2. Outstanding questions that can be investigated using invertebrate systems.**

- How does prior infection history modify different disease-related traits?
- In which traits does heterogeneity matter most for epidemic outcomes?
- How much genetic variation is there in the extent to which prior infection modifies important disease-related traits, and what genes control this variation?
- How can we find better ways to measure the extent of heterogeneity in the initial immune state of individuals?
- Does prior infection always increase heterogeneity in the immune state of individuals?
- Under what conditions will increased heterogeneity result in less severe disease outbreaks?
- Can we classify interventions by their effect on target-trait heterogeneity?

A further level of complexity arises from genetic variation in the expression of each disease-related trait, which also affects the population dynamics of infection [6,18]. An important outstanding question is therefore how much genetic variation there is in the extent to which prior infection modifies important disease-related traits [60]. A full understanding of the epidemiological effects of prior exposure on epidemic outcomes requires a comprehensive assessment of its effects on multiple host traits and measuring the phenotypic and genetic variance in the response to priming present in natural populations [20,24,25]. Here too, insect model systems may prove key. For example, genetic variation in contact rates, pathogen shedding and infectious period were experimentally determined in *Drosophila* infected with Drosophila C Virus [6,53]. The experimental distributions for these traits (measured in males and females of multiple genetic backgrounds) could then be used to parameterize a stochastic epidemiological network model [18]. This approach yielded insights into the relative contribution of each genetic background and sex to the severity and duration of simulated epidemics and revealed that reducing heterogeneity in contact rates (assuming the mean contact rate for all individuals) resulted in epidemic fadeout [18]. By contrast, assuming the mean shedding value for all individuals predicted epidemics that were more severe than would be predicted under a scenario of heterogeneous shedding. This type of detailed data on trait-based heterogeneity is feasible to collect in insect systems to test epidemiological predictions.

Another key aspect that invertebrate systems can powerfully inform is in estimating how infection history can modify important epidemiological parameters, such as the susceptibility to infection. Beyond focusing on the mean susceptibility, understanding how prior infection modifies the heterogeneity in susceptibility—that is, the shape of distribution in susceptibility among all the individuals in a population—is key to epidemiological predictions [1,45,54,64,65]. Previous work in invertebrates such as *Daphnia* [64] and *Drosophila* [65] show that this is feasible by exposing individuals to a wide range of exposure doses to obtain a distribution of susceptibility. The *Daphnia* example is especially noteworthy, as it tested the effect of maternal exposure history on the distribution of offspring susceptibility to the same pathogen. Maternal exposure to the pathogen *Pasteuria ramosa* did not alter the mean susceptibility of offspring but increased the variance in their susceptibility, indicating a broader range of susceptibility within the offspring pool. These examples highlight the power in invertebrate model systems in estimating fundamental epidemiological parameters, and how infection history alters these parameters. Furthermore, these important insights could inform future studies of immune memory and infection history in vertebrates. Where invertebrates have a substantial advantage relative to most vertebrate models is in the potential to combine mechanistic studies at the individual level with studies at the population level [18,20,54,66]. Invertebrates make it possible not only to investigate the immunological responses to priming but, due to the ability to maintain and track hundreds of individuals, also to experimentally dissect their epidemiological consequences.

## Conclusions

Infection history holds a pivotal role in generating individual immune heterogeneity in both vertebrate and inveterate hosts, with important epidemiological consequences. Empirical approaches that link individual-level immune variation to population-level disease dynamics, particularly those that use established model systems of infection, offer promising avenues for addressing many existing theoretical predictions. This integrative approach can improve our understanding of disease ecology, potentially informing public health strategies for disease management and prevention.

## Abbreviations

AMP: antimicrobial peptides
PIM: post-infection mortality
PRR: pattern recognition receptors
SIR: susceptible–infectious–recovered
SPI: susceptible–primed–infectious
